# 3D Printing of a Porous Zn-1Mg-0.1Sr Alloy Scaffold: A Study on Mechanical Properties, Degradability, and Biosafety

**DOI:** 10.3390/jfb15040109

**Published:** 2024-04-18

**Authors:** Xiangyu Cao, Xinguang Wang, Jiazheng Chen, Xiao Geng, Hua Tian

**Affiliations:** 1 Department of Orthopedics, Peking University Third Hospital, Beijing 100191, China; caoxiangyu@bjmu.edu.cn (X.C.); wangxinguang@bjmu.edu.com (X.W.); chenjiazheng@bjmu.edu.cn (J.C.); 2 Engineering Research Center of Bone and Joint Precision Medicine, Ministry of Education, Beijing 100191, China

**Keywords:** selective laser melting, 3D printing, porous Zn-1Mg-0.1Sr alloy scaffold, biodegradable materials, microstructure, biosafety, bone implant material

## Abstract

In recent years, the use of zinc (Zn) alloys as degradable metal materials has attracted considerable attention in the field of biomedical bone implant materials. This study investigates the fabrication of porous scaffolds using a Zn-1Mg-0.1Sr alloy through a three-dimensional (3D) printing technique, selective laser melting (SLM). The results showed that the porous Zn-1Mg-0.1Sr alloy scaffold featured a microporous structure and exhibited a compressive strength (CS) of 33.71 ± 2.51 MPa, a yield strength (YS) of 27.88 ± 1.58 MPa, and an elastic modulus (E) of 2.3 ± 0.8 GPa. During the immersion experiments, the immersion solution showed a concentration of 2.14 ± 0.82 mg/L for Zn^2+^ and 0.34 ± 0.14 mg/L for Sr^2+^, with an average pH of 7.61 ± 0.09. The porous Zn-1Mg-0.1Sr alloy demonstrated a weight loss of 12.82 ± 0.55% and a corrosion degradation rate of 0.36 ± 0.01 mm/year in 14 days. The Cell Counting Kit-8 (CCK-8) assay was used to check the viability of the cells. The results showed that the 10% and 20% extracts significantly increased the activity of osteoblast precursor cells (MC3T3-E1), with a cytotoxicity grade of 0, which indicates safety and non-toxicity. In summary, the porous Zn-1Mg-0.1Sr alloy scaffold exhibits outstanding mechanical properties, an appropriate degradation rate, and favorable biosafety, making it an ideal candidate for degradable metal bone implants.

## 1. Introduction

Bone defects resulting from bone tumor resection, joint revision, traffic accidents, and sports injuries have become common diseases in recent years. These defects present significant clinical challenges, often leading to severe complications such as bone nonunion, atrophy, or deformity [1]. Traditionally, large bone defects are treated with implants made from materials like stainless steel and titanium alloys, which are preferred for their high mechanical strength. However, their higher elastic modulus compared to human bone often results in a stress-shielding effect, which can inhibit new bone growth and lead to complications such as osteoporosis. Moreover, these materials cannot degrade in vivo, which requires surgical removal [2]. Consequently, there has been a growing research interest in developing degradable metallic materials for bone implantation in recent years.

Currently, degradable metallic materials primarily include magnesium (Mg) alloys, iron (Fe) alloys, and zinc (Zn) alloys [3]. Mg alloys degrade rapidly and produce hydrogen (H_2_), which can inhibit tissue growth and healing [4]. Fe alloys degrade slowly, but the formation of cytotoxic Fe(OH)_3_ and the magnetic properties hindering MRI examinations limit their clinical use [5]. Zn alloys degrade at a rate between Mg and Fe, offering more appropriate degradation profiles that are beneficial for bone tissue growth and repair [6]. Li et al. [7] prepared porous Zn using selective laser melting (SLM), which exhibited certain degradation properties and good biocompatibility but relatively poor mechanical performance. Liu et al. [8] used the alloy Zn with appropriate amounts of Mg and Sr elements to produce the Zn-Mg-Sr alloy. The alloy has a finer grain size and a lamellar structure with phases of Zn and MgZn_2_, Mg_2_Zn_11_, and SrZn_13_ eutectic in its matrix, which enhances the mechanical properties of Zn by grain refinement strengthening and solid solution strengthening. Li et al. [9] found that although the Zn alloy possesses certain degradation properties, excessive release of Zn^2+^ in degradation can cause damage to surrounding tissues and organs. The cancellous bone in the skeleton is composed of numerous sponge-like trabeculae, with compressive strength ranging from 2 to 12 MPa, elastic modulus from 0.02 to 3.7 GPa, and porosity from 50% to 90%. This highly porous network structure facilitates the transport of nutrients and the metabolism of substances in bone [10]. Bone defect repair materials require both certain mechanical strength and osteogenic properties. Mg and Sr ions have been reported to enhance the activity of osteoblasts and promote bone tissue growth [11,12]. Adding Mg and Sr elements to Zn can improve its mechanical and biological performance simultaneously. Currently, traditional casting and powder-forming methods are predominantly used for processing Zn alloys. However, these traditional processing methods are not able to fabricate interconnected porous structures that are necessary for bone defect regeneration [13,14]. SLM, which involves computer software-assisted design of three-dimensional solid models, utilizes laser beams to rapidly scan and heat metal powder materials for melting and solidification. This technology provides unprecedented opportunities for the free-form design and fabrication of complex-shaped porous bone implants with excellent mechanical properties and a high surface finish [7,15,16].

To enhance the mechanical strength of the Zn alloy and reduce the damage caused by the excessive release of Zn^2+^ to surrounding tissues, we mimicked the structural characteristics of cancellous bone and designed a minimal surface gyroid structure model. By adding Mg and Sr elements to the Zn alloy and utilizing optimized SLM process parameters, we 3D printed porous Zn-1Mg-0.1Sr alloy scaffolds. Subsequently, we conducted comprehensive research on their microstructure, mechanical properties, degradation behavior, and biocompatibility, aiming to provide a theoretical basis for the development of clinically degradable bone implant metal materials.

## 2. Materials and Methods

### 2.1. Preparation of Porous Zn-1Mg-0.1Sr Alloy Scaffold

**Figure 1:**
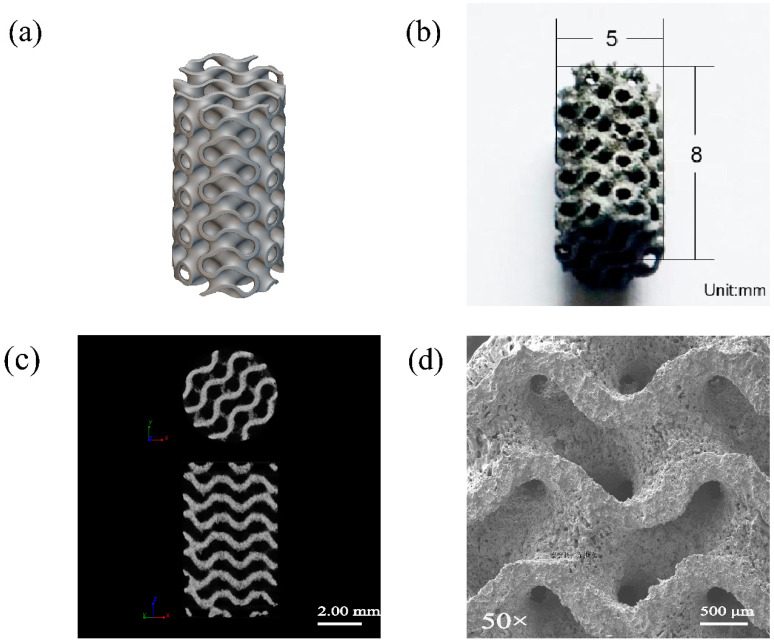
Morphology of the porous Zn-1Mg-0.1Sr alloy scaffold: (**a**) gyroid model; (**b**) microscopic morphology; (**c**) micro-CT image; and (**d**) SEM image.

Based on a dimension of 2 × 2 × 2 mm and a strut thickness of 0.31 mm, a unit cell was designed and iterated to form a minimum surface gyroid structure model (Figure 1a) [7]. The porous Zn-1Mg-0.1Sr alloy scaffold was fabricated using SLM 125 (SLM Solution, Germany). The alloy powder, composed of 98.78 wt.% Zn, 1.09 wt.% Mg, and 0.13 wt.% Sr, was loaded into the machine’s cartridge. The fabrication process was optimized with the following parameters: laser power *P* (70 W), scan speed *V* (600 mm/s), and scan distance *H* (90 μm). The oxygen content within the molding chamber was maintained below 30 ppm. Post-fabrication, the scaffolds were thoroughly cleaned with anhydrous ethanol and distilled water using ultrasonic agitation for 10 min. Acid washing was conducted using anhydrous ethanol containing 1% hydrochloric acid and 1% nitric acid. Subsequently, the scaffolds were ultrasonically cleaned in anhydrous ethanol for 25 min and then dried in a blast drying oven for 60 min. The scaffolds were then stored under sealed conditions.

### 2.2. Micro-Structural Characterization and Composition Analysis of Porous Zn-1Mg-0.1Sr Alloy Scaffolds

The stored porous Zn-1Mg-0.1Sr alloy scaffold was cleaned with anhydrous ethanol and dried in a drying oven for 60 min. The porous Zn-1Mg-0.1Sr alloy scaffolds were scanned using Micro-Computed Tomography (Micro-CT, SkysCan2211, Bruker, Germany), with a tube current of 150 μA, a tube voltage of 120 kV, and a resolution of 4.3 μm^3^. First, the images were locally thresholded over a range of 105 to 265 for the Zn-1Mg-0.1Sr alloy material. Then, the regions of interest (ROIs) (diameter: 5 mm) were outlined. And the pore size, average strut thickness, and porosity of the porous Zn-1Mg-0.1Sr were calculated using BoneJ (a plugin of FIJI) [7]. The microstructure was examined using the scanning electron microscope (SEM) Regulus8100 (Hitachi, Japan). The chemical elemental composition of the scaffold was determined using an energy dispersive spectrometer (EDS) attached to the SEM. Additionally, the phase composition of the alloy scaffold was analyzed using an X-ray diffractometer (TTRⅢ, Rigaku, Japan).

### 2.3. Mechanical Properties of Porous Zn-1Mg-0.1Sr Alloy Scaffolds

Considering that bone defect implant materials are primarily subjected to compressive loads in vivo, this study focuses primarily on the compressive properties of porous Zn-1Mg-0.1Sr alloy scaffolds.

The universal mechanical test machine Instron-5569 (Instron, Norwood, MA, USA) was utilized for the compression tests on the porous Zn-1Mg-0.1Sr alloy scaffolds. Porous, pure Zn scaffolds were used as a control group for comparison. These porous pure Zn alloy scaffolds were designed as porous cylindrical rods with a length of 8 mm, a diameter of 5 mm, and a porosity level of 70 ± 2%. For each group, three parallel specimens were used. The compression rate was set at 1 mm/min. Controlled load testing was conducted to generate stress–strain curves for the alloy scaffolds. The compressive strength (CS) was calculated based on the equivalent area of the porous structure. The yield strength (YS) was determined from the stress value corresponding to 0.2% strain, representing the compressive yield strength. The elastic modulus (E) was ascertained by calculating the slope of the stress–strain curve during the elastic deformation phase. 

### 2.4. Degradation Performance of Porous Zn-1Mg-0.1Sr Alloy Scaffolds

The degradation performance of porous Zn-1Mg-0.1Sr alloy scaffolds was assessed through an immersion test. This test was designed to observe the corrosion degradation morphology, analyze the composition of corrosion products, measure ion release and pH value changes in the immersion solution, and calculate both the percentage weight loss and the corrosion degradation rate of the alloy scaffolds.

The porous Zn-1Mg-0.1Sr alloy scaffolds were precisely weighed using the electronic balance BS210 (Sartorius, Germany). In compliance with the ASTMG31-72 metal corrosion standard [17], the scaffolds were immersed in simulated body fluid (SBF) with a pH of 7.40 after autoclave sterilization [18]. The immersion fluid was replaced every 48 h to simulate the environment in vivo. The immersion tests were conducted in three parallel sets over 14 days. After the immersion test, the samples were air-dried at room temperature without cleaning the degradation products on the surface. The morphology of the scaffold was examined using SEM. The chemical composition of the scaffold surfaces was determined by EDS. The phase composition was acquired by XRD. An inductively coupled plasma optical emission spectrometer (ICP-OES, Agilent, Santa Clara, CA, USA) was used to measure the amounts of Zn^2+^ and Sr^2+^ in the immersion solution. The pH change in the immersion solution was measured with the pH meter PHS-3Cb (Mettler Toledo, Columbus, OH, USA).

After conducting SEM, EDS, and XRD scanning analysis on the post-immersion porous Zn-1Mg-0.1Sr alloy scaffolds, the alloy scaffolds were treated in a 100 g/L ammonium chloride solution at 70 °C to remove surface corrosion products and subsequently reweighed. The weight loss percentage (M%) was calculated using the formula M% = (M_1_ − M_2_)/M_1_ × 100%, where M_1_ is the initial mass of the scaffolds (g) and M_2_ is the mass post-corrosion (g). The corrosion degradation rate (V, in mm/year) was determined using V = K × (M_1_ − M_2_)/A × T × D, where K is a constant (8.76 × 10^4^), A is the surface area (cm^2^), T is the immersion time (h), and D is the material density (g/cm^3^) [19].

### 2.5. Biosafety of Porous Zn-1Mg-0.1Sr Alloy Scaffolds

Biosafety is a paramount consideration for bone implant materials in clinical medicine. In compliance with ISO10993-12:2012 standards [20], the scaffolds were immersed in SBF for three days to produce a 100% leachate. Since the cytotoxicity of Zn is comparable to that of Mg, the extract had to be diluted 6–10 times to meet the international safety standards [21]. The 100% extract was diluted to 50%, 20%, and 10% concentrations using SBF. The effects of alloy scaffold extracts with different concentrations on the activity and proliferation of osteoblast precursor cells (MC3T3-E1, ATCC CRL-2594, Manassas, VA, USA) were further studied to evaluate biosafety.

The study established a negative control group, a positive control group, and an experimental group. The experimental group was further subdivided with 100%, 50%, 20%, and 10% extract concentrations. Each group had six parallel samples, with three dedicated to cell activity and quantification and three for cell live/dead staining. MC3T3-E1 cells, cultured in Dulbecco’s Modified Eagle’s Medium (DMEM, Gibco, Norristown, PA, USA), were seeded into 48-well plates at a density of 3 × 10^3^ cells/well. The negative control group received DMEM; the positive control group received DMEM with 10% dimethyl sulfoxide; and the experimental groups received varying concentrations of the extracts. The cells were incubated for 1, 3, and 5 days at 37 °C in a 5% CO_2_ incubator, with the medium refreshed every 24 h. 

The viability and proliferation of MC3T3-E1 cells were assessed using a Cell Counting Kit-8 (CCK-8, Shimadzu, Japan) [22]. A mixture of CCK-8 and DMEM (1:10) was added to each well, followed by incubation for one hour. The optical density (OD) at 450 nm was measured using a multi-mode plate reader (Envision, Perkin Elmer, Waltham, MA, USA). The relative growth rate (RGR) was calculated as RGR = (OD value of experimental group/OD value of negative control group) × 100% [23]. Cytotoxicity grading was based on RGR: grade 0 (100% or higher; safe and non-toxic), grade 1 (75–99%; minimally toxic), grade 2 (50–74%; mildly toxic), grade 3 (25–49%; moderately toxic), and grade 4 (1–24%; severely toxic).

The live/dead cell assay kit (Solarbio, Beijing, China) was used to assess the viability of MC3T3-E1 cells exposed to various extract concentrations [24]. In light-protected conditions, 500 μL of staining agent solutions were added to each well and incubated for 20 min. The cells were observed under a fluorescence microscope (Optiphot-2, Nikon, Japan). Live cells exhibited green fluorescence, while dead cells exhibited red fluorescence.

### 2.6. Statistical Analysis

The data were analyzed using SPSS 17.0 software. A one-way analysis of variance (ANOVA) followed by the least significant difference (LSD) test was used to compare the means of multiple groups. A value of *p* < 0.05 was considered to have statistical significance.

## 3. Results

### 3.1. Microstructure of Porous Zn-1Mg-0.1Sr Alloy Scaffolds

**Figure 2:**
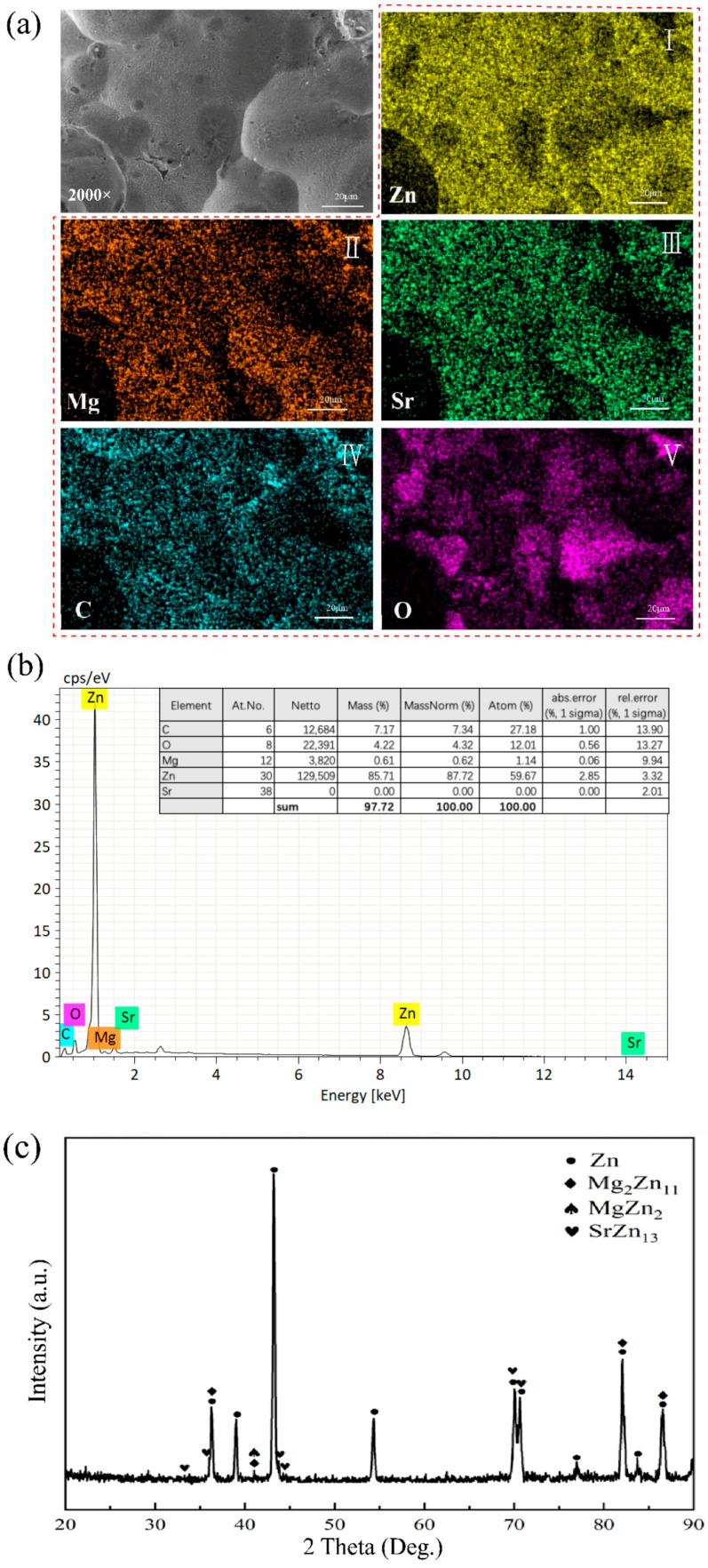
EDS and XRD analysis of the porous Zn-1Mg-0.1Sr alloy scaffold: (**a**,**b**) EDS analysis image; (**c**) XRD pattern.

The SLM technique was utilized to 3D print the porous Zn-1Mg-0.1Sr alloy scaffolds. These scaffolds were characterized by a cylindrical structure with a length of 8 mm and a diameter of 5 mm. The average strut size and pore size of the as-built scaffolds were 335 ± 2 μm (design value = 310 μm) and 550 ± 27 μm (design value = 500 μm), respectively. The porosity was calculated to be 70 ± 2%. The scaffolds exhibited a relatively uniform distribution of holes. Notably, there is considerable connectivity between these holes, creating a three-dimensional scaffold structure (Figure 1b–d). EDS analysis revealed a matrix composition consisting primarily of Zn, C, O, Mg, and Sr. Zn is the dominant element (Figure 2a,b). XRD analysis indicated that the matrix of the alloy scaffolds is predominantly a eutectic structure composed of primary dendritic α-Zn and the secondary phase Mg_2_Zn_11_. Additionally, there are some intermediate compounds formed by the eutectic reaction of the MgZn_2_ phase. There is also a minor presence of irregular SrZn_13_ phases precipitated through the eutectic reaction of Sr and Zn (Figure 2c).

### 3.2. Mechanical Properties of Porous Zn-1Mg-0.1Sr Alloy Scaffolds

The compression test revealed that porous Zn-1Mg-0.1Sr alloy scaffolds exhibited a uniform distribution of holes and intact pore walls under no stress conditions. During the compression process, the holes demonstrated shrinkage, deformation, and collapse, with some pores collapsing and merging completely. This suggests that porous Zn-1Mg-0.1Sr alloy scaffolds possess substantial plastic strain capacity and are unlikely to be catastrophically damaged in clinical applications.

**Figure 3:**
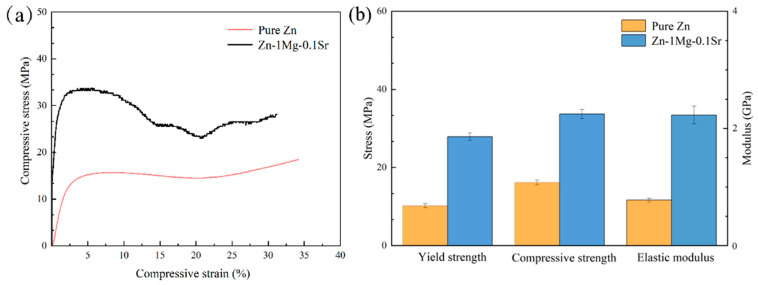
Compression tests: (**a**) stress–strain curves of porous Zn-1Mg-0.1Sr and pure Zn alloy scaffolds; (**b**) compressive strength of porous Zn-1Mg-0.1Sr and pure Zn alloy scaffolds.

The stress–strain curves and compression properties of both porous Zn-1Mg-0.1Sr and porous Zn alloy scaffolds were acquired through compression tests (Figure 3a,b). The CS of the Zn-1Mg-0.1Sr alloy scaffold was recorded as 33.71 ± 2.51 MPa, the YS was 27.88 ± 1.58 MPa, and the E was 2.3 ± 0.8 GPa. Comparatively, the porous pure Zn alloy scaffold showed a CS of 16.17 ± 0.71 MPa, a YS of 10.21 ± 0.38 MPa, and an E of 0.8 ± 0.1 GPa. These results indicate that porous Zn-1Mg-0.1Sr alloy scaffolds have higher compressive, yield strengths, and elastic moduli compared to porous Zn alloy scaffolds.

### 3.3. Degradation Performance of Porous Zn-1Mg-0.1Sr Alloy Scaffolds

**Figure 4:**
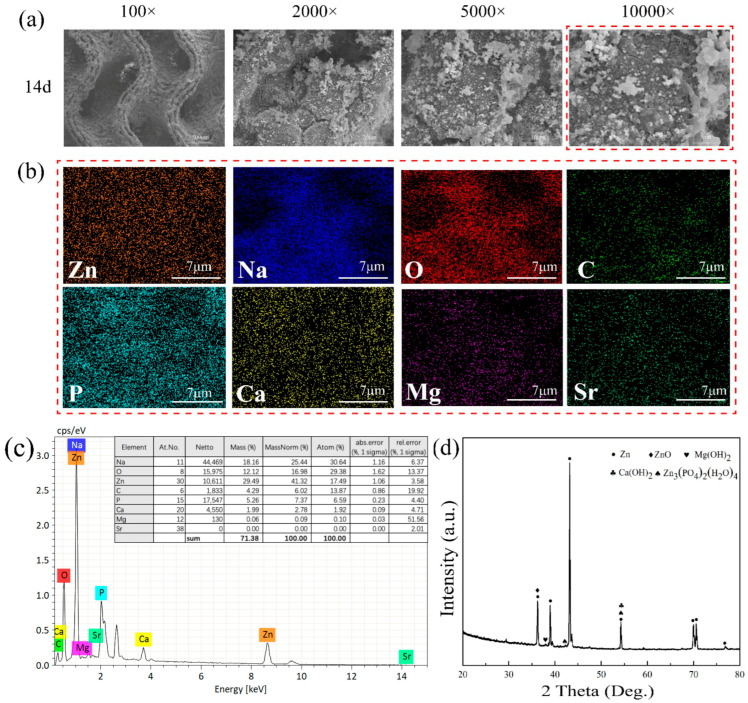
SEM images, EDS analysis, and XRD analysis of the porous Zn-1Mg-0.1Sr alloy scaffold after immersion in SBF for various durations: (**a**) SEM images; (**b**,**c**) EDS analysis images; and (**d**) XRD pattern.

After the porous Zn-1Mg-0.1Sr alloy scaffold was immersed for 14 d, a progressively thicker corrosion layer formed on the scaffold’s surface. This led to several holes in the scaffold becoming filled with degradation products (Figure 4a).

EDS analysis of the corrosion degradation products revealed a composition primarily of Zn, O, Na, P, Ca, C, Mg, and Sr after immersion for 14 d. Zn was the most dominant element, with Mg and Sr less abundant from 1 d to 14 d (Figure 4b,c). XRD analysis showed that the degradation products mainly consisted of Zn, ZnO, Ca(OH)_2_, Mg(OH)_2_, and phosphate Zn_3_(PO_4_)_2_(H_2_O)_4_. As the immersion time increased, the insoluble Ca-P and Zn degradation products gradually increased, covering the alloy scaffold’s surface (Figure 4d).

**Figure 5:**
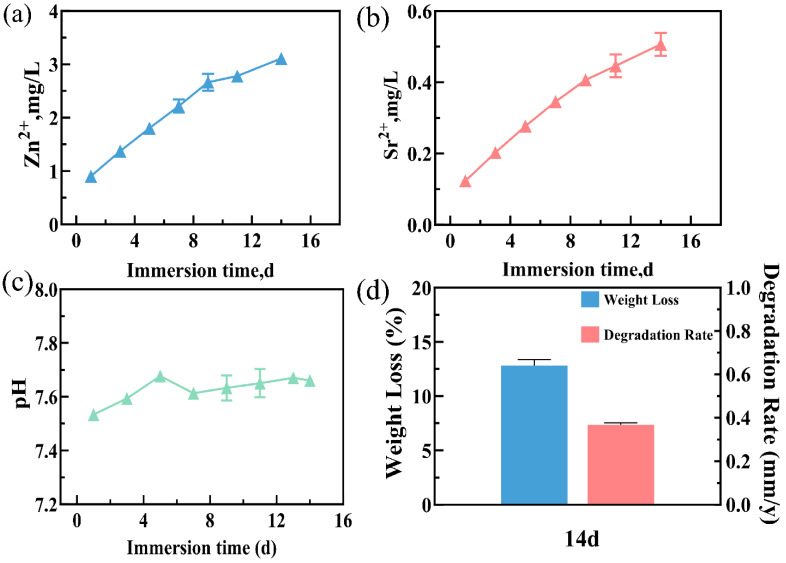
Porous Zn-1Mg-0.1Sr alloy scaffold in SBF over time: (**a**) Zn^2+^ amount; (**b**) Sr^2+^ amount. (**c**) pH value variation curves; (**d**) corrosion degradation weight loss percentage and corrosion degradation rate.

Since Mg^2+^ was already present in the SBF, this study only measured the concentration changes in Zn^2+^ and Sr^2+^ released from the corrosion degradation. The results showed that the release rate of Zn^2+^ was rapid during 1 d to 14 d, with an average concentration of 2.14 ± 0.82 mg/L. The release rate of Sr^2+^ increased steadily, with an average concentration of 0.34 ± 0.14 mg/L. The release amount of Zn^2+^ was significantly higher than that of Sr^2+^ (Figure 5a,b).

The pH value of the immersion solution increased to 7.68 ± 0.01 at 5 d, then decreased slightly at 7 d, and gradually climbed to 7.67 ± 0.01 at 14 d. The average pH value was 7.61 ± 0.09. In brief, the pH value of the immersion solution increased rapidly within the first five days, and then increased slowly. From 5 d to 14 d, the pH value remained fluctuating at 7.66 ± 0.03 (Figure 5c).

After immersion for 14 d, the alloy scaffold lost 12.82 ± 0.55% of its weight. This weight loss progressively increased with immersion time. The corrosion degradation rate was measured at 0.36 ± 0.01 mm/year after 14 days of immersion. (Figure 5d).

### 3.4. Biosafety of Porous Zn-1Mg-0.1Sr Alloy Scaffolds

**Figure 6:**
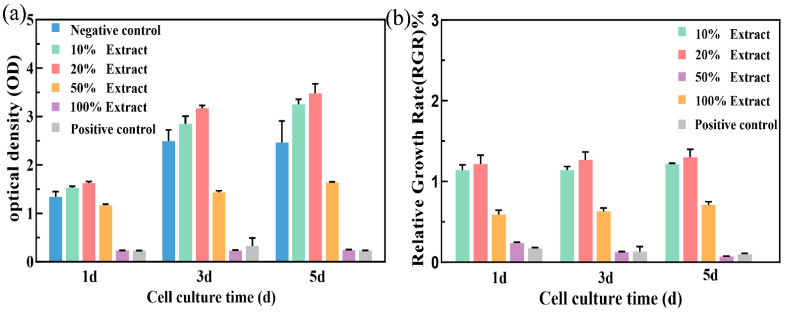
Optical density (OD) and relative growth rate (RGR%) of MC3T3-E1 cells in each experimental group: (**a**) optical density (OD); (**b**) relative growth rate (RGR%).

**Figure 7:**
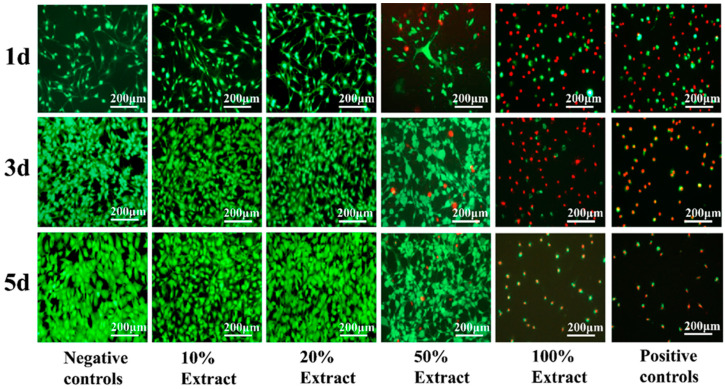
Live/dead fluorescence staining of MC3T3-E1 cells exposed to different concentrations of extracts and for different culture periods.

To assess the biosafety of porous Zn-1Mg-0.1Sr alloy scaffolds, a 100% extract was obtained by immersing the scaffold in SBF for three days. The original 100% extract was diluted with the appropriate amount of SBF to obtain 50%, 20%, and 10% extracts. The CCK-8 was used to evaluate the viability and proliferation of MC3T3-E1 cells cultured with different extract concentrations for 1 d, 3 d, and 5 d (Figure 6a,b). Live and dead cell staining was observed by inverted fluorescence microscopy (Figure 7). Significant differences in optical density (OD) values were observed among the negative control group, positive control group, and the 10%, 20%, 50%, and 100% extract groups (F = 370.082, 249.644, and 594.882, respectively, *p* < 0.01). MC3T3-E1 cells showed better activity in the negative control group and the 10% and 20% extract groups, with a higher number of live cells and a spindle morphology. As the culture time increased, the cell numbers and OD values progressively increased. The cell numbers and OD values peaked in the 20% extract group. The RGR in this group exceeded 100%, indicating cytotoxicity as grade 0 (safe and non-toxic). The 50% extract group showed good cell activity at 1 d, 3 d, and 5 d. But red fluorescence was detected, indicating the presence of dead cells. Over time, the number of live cells gradually increased, leading to an increase in OD values. The RGR in this group ranged from 50% to 74%, indicating the as grade 2 (mild toxicity). In the 100% extract group, MC3T3-E1 cells showed lower activity at all time points, characterized by a sparse distribution and mostly round morphology. The live cells were rare, while the dead cells were abundant. Cell activity, OD values, and RGR decreased significantly with prolonged culture time. The RGR was less than 24%, indicating the cytotoxicity to be grade 4 (severe toxicity). In summary, the 10% and 20% extracts were non-toxic, the 50% extract showed mild toxicity, and the 100% extract exhibited severe toxicity.

## 4. Discussion

### 4.1. Microstructure of Porous Zn-1Mg-0.1Sr Alloy Scaffolds

Currently, there is a great demand for bone defect repair materials. Degradable Zn-based alloys have become a research highlight as novel bone graft materials. Based on a unit cell size of 2 × 2 × 2 mm and a strut thickness of 0.31 mm, we designed a unit cell shape and iterated to form a minimum surface gyroid structure model [7]. Supported by computer software for designing three-dimensional solid models, SLM 3D printing utilizes laser beams to rapidly scan and heat metal powder materials for solidification. This technique enables the fabrication of alloys with excellent mechanical properties, a high surface finish, and complex three-dimensional structures [7,15,16]. In this study, SLM was used to fabricate porous cylindrical structures with a diameter of 5 mm, a length of 8 mm, and a porosity of 70 ± 2%. Several considerations were made to ensure the printing accuracy of the alloy scaffolds. Firstly, high-quality powder is crucial. Because the 3D printing process is a layer-by-layer process, uniform powder deposition and an appropriate thickness per layer are required. The powder should have good sphericity, appropriate particle size, and suitable dryness to facilitate flowability [25]. Secondly, a constant substrate preheating temperature can enhance powder flowability, reduce stress, and prevent cracking [26]. Thirdly, appropriate process parameters, such as laser energy output and scanning speed, were set. Increasing the scanning speed under the same laser power reduces the laser energy output, which may lead to poor fusion between layers and defects such as porosity [25,27]. Furthermore, the oxygen content also influences the properties of Zn alloys. Controlling this content can diminish the oxidation of Zn alloys [28]. Therefore, we optimized the parameters for SLM 3D printing: laser power (P) at 70 W, scanning rate (V) at 600 mm/s, scanning distance (H) at 90 μm, and oxygen content in the building chamber below 30 ppm. This was designed to achieve the desired printing quality and to ensure the alloy scaffolds can meet the requirements.

Previous studies have found that Zn-Mg-Sr bulk alloys exhibit excellent mechanical properties, appropriate degradation rates, and good biocompatibility [8]. The porous Zn-1Mg-0.1Sr alloy scaffold in this study exhibits a porous structure with a relatively uniform distribution of micropores. Certain interconnections between the pores help form a three-dimensional scaffolding structure akin to the human cancellous bone [10]. Once implanted into the human body, new bone tissues, blood vessels, and nerves can infiltrate the pores, creating a multidimensional crossover structure. This not only enhances the mechanical strength of the alloy material but also fosters an ideal environment for tissue and cell growth. It promotes cell adhesion, proliferation, and differentiation, facilitates nutrient and metabolic exchange, and supports tissue growth and repair [10,29]. While the correlation between porosity and bone tissue healing remains controversial, it is generally believed that pore sizes ranging from 600 to 1200 μm and porosities between 70% and 90% are more conducive [30,31]. Porosity is a critical factor affecting the mechanical and degradation properties of porous metal materials, as increased porosity can lead to a decrease in the compressive strength and elastic modulus [32]. When designing porous metallic materials, the mechanical properties should be consistent with the requirements of the human bone restoration site. EDS analysis of the porous Zn-1Mg-0.1Sr alloy scaffold revealed that the scaffold matrix mainly contains the elements Zn, C, O, Mg, and Sr, with Zn being the most abundant element. XRD analysis showed that the primary phase in the scaffold matrix is the primary dendritic α-Zn phase, accompanied by secondary phases such as Mg_2_Zn_11_, MgZn_2_, and SrZn_13_, which act as solid solution reinforcers. These secondary phases result in a finer grain size and lamellar structure, thereby enhancing the mechanical properties of the Zn alloy [33]. Therefore, by incorporating Mg and Sr elements into the Zn alloy and employing optimized SLM process parameters, we were able to 3D print porous Zn-1Mg-0.1Sr alloy scaffolds with a porosity of 70 ± 2%. Subsequently, we investigated their mechanical properties, degradation behavior, and biocompatibility, laying a theoretical foundation for clinically degradable bone implant metal materials.

### 4.2. Mechanical Properties of Porous Zn-1Mg-0.1Sr Alloy Scaffolds

Mechanical properties are crucial for biodegradable metallic materials used as bone implants. These materials must possess mechanical properties that align with the strength of the local tissue and environment, especially considering that materials intended for filling bone defects predominantly endure compressive loading in vivo. Consequently, this study focused on the compressive properties of porous Zn-1Mg-0.1Sr alloy scaffolds.

Compression testing revealed that the porous Zn-1Mg-0.1Sr alloy scaffolds maintained a uniform distribution of holes and intact pore walls under no stress. However, during the compression process, the holes exhibited shrinkage, deformation, and collapse, with some pores completely collapsing and merging. The Zn-1Mg-0.1Sr scaffolds possess considerable plastic strain capacity and could endure substantial stress without catastrophic damage.

The CS of the porous Zn-1Mg-0.1Sr alloy scaffold obtained from compression tests is 33.71 ± 2.51 MPa, with a YS of 27.88 ± 1.58 MPa. These values are significantly higher compared to the CS of 16.17 ± 0.71 MPa and YS of 10.21 ± 0.38 MPa for the porous Zn scaffold. Li et al. [7] found that the YS of porous Zn scaffolds was 10.8 MPa with a pore size of 550 ± 38 μm and a porosity of 62 ± 2%, indicating that the strength of porous Zn scaffolds is low. Similarly, Fan et al. [31] found that 3D-printed porous Zn scaffolds with 67% porosity had low YS (11 MPa) and low stiffness strength (0.8 GPa). Although the addition of Mg and Sr contributed to a significant improvement in mechanical properties, the porous Zn-1Mg-0.1Sr alloy scaffolds in this study still exhibited much lower strength compared to that of the bulk Zn-Mg-Sr alloys (YS: 196.84 ± 13.20 MPa) [34]. The enhanced mechanical properties of the Zn-1Mg-0.1Sr scaffolds were attributed to two factors. Firstly, the optimization of SLM processing parameters ensured high alloy density and minimal internal defects [35]. Secondly, the integration of Mg and Sr with Zn in the alloy matrix formed secondary phases such as Mg_2_Zn_11_, MgZn_2_, and SrZn_13_. These secondary phases contributed to solid solution strengthening [12]. Vojtech et al. [36] found that Mg was shown to substantially increase the tensile strength and elongation of Zn alloys. This effect peaks at around 1% Mg content. The Mg content in the Zn-1Mg-0.1Sr alloy was consistent with this. As the porosity of the alloy increases, its mechanical strength and elastic modulus decrease [32]. Although the CS of this porous Zn-1Mg-0.1Sr alloy scaffold was only 33.71 ± 2.51 MPa, it significantly exceeded the CS of human cancellous bone (2–12 MPa) [10,37]. The elastic modulus of the scaffold was 2.3 ± 0.8 GPa; it was also considerably lower than that of human cortical bone (10–30 GPa) but was comparable to the elastic modulus of human cancellous bone (0.02–3.7 GPa) [10,38]. Thus, the porous Zn-1Mg-0.1Sr alloy scaffold effectively reduced the elastic modulus while maintaining adequate mechanical strength. This reduction in the modulus can help avoid the stress shielding effect and promote surrounding bone tissue growth, which helps meet the requirements for bone defect implantation materials.

### 4.3. Degradation Performance of Porous Zn-1Mg-0.1Sr Alloy Scaffolds

A key characteristic of degradable metallic materials is their degradation performance. A comprehensive evaluation of this performance in a simulated human environment is crucial to ensuring the stability of porous scaffold materials after implantation.

With prolonged immersion, white granular degradation products accumulated on the surface of the porous Zn-1Mg-0.1Sr alloy scaffold. This accumulation resulted in a progressively thicker corrosion layer, with some pores gradually becoming filled with degradation products. Additionally, the development of pits and micro-cracks was observed on the scaffold’s surface. This is consistent with the findings of Li et al. [7]. EDS identified these degradation products as consisting mainly of the elements Zn, O, Na, P, Ca, C, Mg, and Sr. Compared with the EDS results before degradation, it is likely that the Na, P, and Ca elements originated from the SBF. The high Na concentration and low Mg concentration may be due to the irregular distribution of degradation products on the surface. XRD analysis further indicated that the main constituents were Zn, ZnO, Ca(OH)_2,_ Mg(OH)_2_, and phosphate Zn_3_(PO_4_)_2_(H_2_O)_4_. Compared with the XRD results before degradation, these compounds, such as ZnO, Ca(OH)_2_, Mg(OH)_2_, and zinc phosphate Zn_3_(PO_4_)_2_(H_2_O)_4_, are likely degradation products generated from chemical reactions between the porous Zn-1Mg-0.1Sr alloy scaffold and SBF. These findings align with those reported by Hybasek et al. [39]. Although the porous Zn-1Mg-0.1Sr alloy scaffold includes Sr, XRD did not detect Sr-containing degradation products, probably due to the low Sr content. As immersion prolonged, the accumulation of Ca-P and Zn corrosion products increased, while the concentration of OH^−^ decreased, without the production of H_2_ [40]. The expansion coefficient disparity between the degradation products on the surface and the metal matrix induces stress differences, potentially causing cracking or spalling [41]. Furthermore, Cl^−^ in the immersion solution can disrupt the oxide film on the scaffold’s surface, leading to galvanic corrosion, pitting, and micro-cracking [42]. These alterations may initially reduce the mechanical properties of the scaffold. However, as more degradation products progressively cover the surface and pores, the mechanical strength of the scaffold may be enhanced [43]. Since the porous Zn-1Mg-0.1Sr alloy scaffold was only immersed for 14 days, the influence of adhered degradation products on the mechanical properties of the alloy scaffold is probably minimal.

During the initial days of immersion, the release rates of Zn^2+^ and Sr^2+^ from the scaffold were relatively rapid. As the immersion time extended, the release rates of Zn^2+^ and Sr^2+^ gradually increased. Notably, the release of Zn^2+^ significantly exceeded that of Sr^2+^. This phenomenon may be attributed to the corrosion degradation of the porous Zn-1Mg-0.1Sr alloy scaffold, wherein Cl^−^ in the immersion solution disrupted the oxide film and led to galvanic corrosion. Additionally, due to the more negative potentials of Mg and Sr, the preferential dissolve accelerated the release rates of Zn^2+^ and Sr^2+^ ions [44]. As degradation products accumulated within the pores of the scaffold, the overall degradation rate of the scaffold decelerated, and the release rates of Zn^2+^ and Sr^2+^ exhibited a slow, increasing trend.

The pH value of the porous Zn-1Mg-0.1Sr alloy scaffolds rapidly increased during the initial 1 d and 5 d of immersion. This was mainly due to the rapid degradation and the consequent excessive formation of OH^−^. A slight decrease in pH was noted on day 7, followed by a gradual increase. This fluctuation might be associated with the reaction between OH^−^ and Zn^2+^, leading to the increasingly abundant Zn(OH)_2_, ZnO, and other products [45]. The pH value during the degradation process is dynamic and does not lead to a severe local alkaline environment.

When immersed in SBF for 14 d, the weight loss percentage of the porous Zn-1Mg-0.1Sr alloy scaffolds was 12.82 ± 0.55%, and the corrosion degradation rate was 0.36 ± 0.01 mm/year. The weight loss of the alloy scaffold in SBF increased over time, while the degradation rate slowed down with the prolonged soaking time. Li et al. [7] found that the degradation rate of addictively manufactured porous Zn scaffolds was 0.07 mm/year in a static environment and 0.13 mm/year in a dynamic environment. Others reported that the degradation rate of addictively manufactured porous Zn scaffolds was 0.011–0.084 mm/year [46,47]. The degradation rate of porous Zn-1Mg-0.1Sr alloy scaffolds in this study is faster than that of porous Zn scaffolds, which could be attributed to the Mg and Sr alloying. Notably, the corrosion potential of Zn is −0.76 V, while that of Mg and Sr is lower. The addition of Mg and Sr to the Zn alloy can accelerate the corrosion degradation rate [48]. Additionally, due to grain refinement, the rapid cooling in the SLM process increases the grain boundary area in the Zn alloy. The second phases, Mg_2_Zn_11_, MgZn_2_, and SrZn_13_, participated in micro-galvanic corrosion and impacted the degradation rate [49]. The degradation rate of high-porosity alloy scaffolds is notably faster than that of bulk alloys [50]. The degradation products on the surface of scaffolds also had an influence on this rate [51]. Bone implant materials are typically required to maintain functionality for 3 to 6 months [52]. The degradation rate of alloy materials in vitro should also be less than 0.5 mm/year [53]. Our porous Zn-1Mg-0.1Sr alloy scaffolds showed an average degradation rate of 0.36 ± 0.01 mm/year, which is already below the suggested in vitro degradation rates of alloy materials. This result confirmed that the porous Zn-1Mg-0.1Sr alloy scaffold met the requirements for bone implant materials.

### 4.4. Biosafety of Porous Zn-1Mg-0.1Sr Alloy Scaffolds

The biosafety of degradable metallic implants predominantly depends on the ion concentration and degradation products released. It is acknowledged that a low concentration of Zn^2+^ can stimulate cell activity and a high concentration poses certain cytotoxic effects [9], while there are discrepancies in the results due to different culture media and cell types [54,55]. 

In this study, the results showed that MC3T3-E1 cells cultured in 10% and 20% extract groups exhibited good bioactivity. The number of live cells marked by green fluorescence was higher in the 20% extract group, which indicated that 10% and 20% extracts had a beneficial effect on promoting cell activity and proliferation. The activity of MC3T3-E1 cells in the 50% extract group was also relatively good, but dead cells marked with red fluorescence could be detected, indicating that the 50% extract had a substantial cytotoxic effect. In the 100% extract group, the activity of MC3T3-E1 cells was bad, accompanied by sparse cells and mostly round morphology. The count of live cells was notably low, while dead cells marked by red fluorescence staining were abundant. With the prolongation of culture time, the cell activity further decreased, indicating that the 100% extract was cytotoxic. The concentration of Zn^2+^ within the exact might be beyond the tolerance of cells. Overall, the 10% and 20% extracts showed no toxicity, the 50% extract exhibited low toxicity, and the 100% extract presented high toxicity, a trend aligning with the findings of Capek et al. [50]. Zn, an essential trace element, is known to promote osteoblast growth, inhibit osteoclast differentiation, and enhance bone tissue healing [56]. Mg, which is essential for normal metabolism, can enhance cell activity, promote adhesion and proliferation, and aid in the healing of bone tissue at appropriate levels [11]. Sr, a bone-supporting element, has been shown to promote cell differentiation and new bone formation, although the low concentration of Sr^2+^ results in a minimal effect on cell activity [12]. Hernandez et al. [57] observed that the Zn alloy exhibits significant cytotoxic effects in vitro, but the toxicity is markedly reduced in vivo. This reduction may be due to protein absorption, body fluid dynamics, and the possibility that degradation products may be recycled and excreted in feces and urine [58]. The human body fluid environment comprises numerous inorganic salts, oxygen, hormones, cell metabolites, enzymes, antibodies, and other mediators. Consequently, the corrosion and degradation of Zn alloy materials in vivo are intricate processes [59]. Therefore, in vitro tests are crucial in assessing the biosafety of Zn alloy materials prior to their clinical application.

### 4.5. Limitations and Prospects

This study investigated the microstructure, mechanical properties, degradation behavior, and biocompatibility of porous Zn-1Mg-0.1Sr alloy scaffolds in vitro. To better understand the degradation mechanisms, more detailed characterization techniques such as X-ray photoelectron spectroscopy (XPS) should be performed in the future. Additionally, the biological performance of the alloy scaffolds in animal models has not been studied. In future studies, we will employ various approaches to obtain detailed information on the surface chemical composition of the alloy scaffolds and investigate their biological performance in vivo.

The development of Zn-1Mg-0.1Sr alloy scaffolds is aimed at providing bone graft materials for the clinical treatment of bone defects. Therefore, future research should focus on the biomimetic and functionality of porous Zn-1Mg-0.1Sr alloy scaffolds to further improve their comprehensive performance.

## 5. Conclusions

This study utilized SLM-fabricated porous Zn-1Mg-0.1Sr alloy scaffolds and investigated the feasibility of bone implantation as a degradable metal material. The key findings are summarized.

The microporous structure of porous Zn-1Mg-0.1Sr alloy scaffolds allows for inter-pore communication. This facilitates nutrient distribution and promotes the growth of surrounding tissue, which is beneficial for tissue repair. The incorporation of Mg and Sr elements into the scaffold results in grain refinement, which enhances mechanical properties and reduces the elastic modulus. These features enable the scaffold to effectively fill bone defects and avoid the stress shielding effect. Porous Zn-1Mg-0.1Sr alloy scaffolds exhibit a distinct degradation characteristic. This characteristic allows the scaffolds to gradually degrade during bone defect repair, eliminating the need for surgical removal. Furthermore, the scaffolds demonstrate excellent biosafety, which helps promote bone defect repair.

In summary, the SLM 3D-printed porous Zn-1Mg-0.1Sr alloy scaffold possesses favorable mechanical properties, an appropriate degradation rate, and good biosafety. However, its application in the human body requires further validation through in vivo animal experiments. The porous Zn-1Mg-0.1Sr alloy material holds promise as a viable degradable metal material for bone implantation.

## Data Availability

Data sets generated during the current study are available from the corresponding author on reasonable request.

## References

[1] Dilogo I.H., Rahmatika D., Pawitan J.A., Liem I.K., Kurniawati T., Mujadid T.K.F. (2021). Allogeneic umbilical cord-derived mesenchymal stem cells for treating critical-sized bone defects: A translational study. Eur. J. Orthop. Surg. Traumatol..

[2] Li F., Li S., Liu Y., Zhang Z., Li Z. (2022). Current advances in the roles of doped bioactive metal in biodegradable polymer composite scaffolds for bone repair: A mini review. Adv. Eng. Mater..

[3] Kabir H., Munir K., Wen C., Li Y. (2020). Recent research and progress of biodegradable zinc alloys and composites for biomedical applications: Biomechanical and biocorrosion perspectives. Bioact. Mater..

[4] Akbarzadeh F.Z., Ghomi E.R., Ramakrishna S. (2022). Improving the corrosion behavior of magnesium alloys with a focus on AZ91 Mg alloy intended for biomedical application by microstructure modification and coating. Proc. Inst. Mech. Eng. Part H J. Eng. Med..

[5] Li G., Yang H., Zheng Y., Chen X.H., Yang J.A., Zhu D., Ruan L., Takashima K. (2019). Challenges in the use of zinc and its alloys as biodegradable metals: Perspective from biomechanical compatibility. Acta Biomater..

[6] Zhuang Y., Liu Q., Jia G., Li H., Yuan G., Yu H. (2021). A Biomimetic Zinc Alloy Scaffold Coated with Brushite for Enhanced Cranial Bone Regeneration. ACS Biomater. Sci. Eng..

[7] Li Y., Pavanram J., Zhou J., Lietaert K., Taheri P., Li W., San H., Leeflang M.A., Mol J.M.C., Jahr H. (2020). Additively manufactured biodegradable porous zinc. Acta Biomater..

[8] Liu Y., He S., Li Y., Liu Z., Li C., Li J., Miao H., Zhu D., Su L. (2023). In vitro degradation behavior and microstructural evolution of a novel biodegrad able Zn-Mg-Sr alloy during homogenization. J. Mater. Eng. Perform..

[9] Li P., Dai J., Schweizer E., Rupp F., Heiss A., Richter A., Alexander D. (2020). Response of human periosteal cells to degradation products of zinc and its alloy. Mater. Sci. Eng. C.

[10] Falcinelli C., Whyne C. (2020). Image-based finite-element modeling of the human femur. Comput. Methods Biomech. Biomed. Eng..

[11] Zhou H., Liang B., Jiang H., Deng Z., Yu K. (2021). Magnesium-based biomaterials as emerging agents for bone repair and regeneration:From mechanism to application. J. Magnes. Alloys.

[12] Shi Z.Z., Gao X.X., Zhang H.J., Liu X.-F., Li H.-Y., Zhou C., Yin Y.-X., Wang L.-N. (2020). Design biodegradable Zn alloys: Second phases and their significant influences on alloy properties. Bioact. Mater..

[13] Gong H., Wang K., Strich R., Zhou J. (2015). In vitro biodegradation behavior, mechanical properties, and cytotoxicity of biodegradable Zn–Mg alloy. J. Biomed. Mater. Res. Part B Appl. Biomater..

[14] Hwang S., An S., Robles U., Rumpf R.C. (2023). Process parame teropimization for removable partialdenture frame works manufactured by select velaser melting. J. Prost. Dent..

[15] Li P., Dai J., Li Y., Alexander D., Čapek J., Geis-Gerstorfer J., Wan G., Han J., Yu Z., Li A. (2023). Zinc based biodegradable metals for bone repair and regeneration: Bioactivity and molecular mechanisms. Mater. Today Bio.

[16] Carluccio D., Demir A.G., Bermingham M.J., Dargusch M.S. (2020). Challenges and opportunities in the selective laser melting of biodegradable metals for load-bearing bone scaffold applications. Metall. Mater. Trans. A.

[17] Nace A. (2012). Standard Guide for Laboratory Immersion Corrosion Testing of Metals.

[18] Jia B., Yang H., Han Y., Zhang Z., Qu X., Zhuang Y., Wu Q., Zheng Y., Dai K. (2020). In vitro and in vivo studies of Zn-Mn biodegradable metals designed for orthopedic applications. Acta Biomater..

[19] Zhao L., Zhang Z., Song Y., Liu S., Qi Y., Wang X., Cui C. (2016). Mechanical properties and in vitro biodegradation of newly developed porous Zn scaffolds for biomedical applications. Mater. Des..

[20] (2012). Sample Preparation and Reference Materials.

[21] Wang J., Witte F., Xi T., Zheng Y., Yang K., Yang Y., Zhao D., Meng J., Li Y., Li W. (2015). Recommendation for modifying current cytotoxicity testing standards for biodegradable magnesium-based materials. Acta Biomater..

[22] Zhen Z., Liu X., Huang T., Xi T.F., Zheng Y.F. (2015). Hemolysis and cytotoxicity mechanisms of biodegradable magnesium and its alloys. Mater. Sci. Eng. C Mater. Biol. Appl..

[23] Li P., Schille C., Schweizer E., Kimmerle-Müller E., Rupp F., Heiss A., Legner C., Klotz U.E., Geis-Gerstorfer J., Scheideler L. (2019). Selection of extraction medium influences cytotoxicity of zinc and its alloys. Acta Biomater..

[24] Sun Y.H., Zhao Y., Zhao Y.Y., Rong Y.J., Yao R.H., Yao X.H., Chu P.K. (2021). Improving exposure of anodically ordered Ni-Ti-O and corrosion resistance and biological properties of NiTi alloys by substrate electropolishing. Rare Met..

[25] Wen P., Jauer L., Voshage M., Chen Y., Poprawe R., Schleifenbaum J.H. (2018). Densification behavior of pure Zn metal parts produced by selective laser melting for manufacturing biodegradable implants. J. Mater. Process. Technol..

[26] Hu D., Wang Y., Zhang D., Hao L., Jiang J., Li Z., Chen Y. (2015). Experimental investigation on selective laser melting of bulk net-shape pure magnesium. Mater. Manuf. Process..

[27] Li Y., Li W., Bobbert F.S., Lietaert K., Dong J.H., Leeflang M.A., Zhou J., Zadpoor A.A. (2020). Corrosion fatigue behavior of additively manufactured biodegradable porous zinc. Acta Biomater..

[28] Leitz K.H., Grohs C., Singer P., Tabernig B., Plankensteiner A., Kestler H., Sigl L.S. (2018). Fundamental analysis of the influence of powder characteristics in Selective Laser Melting of molybdenum based on a multi-physical simulation model. Int. J. Refract. Met. Hard Mater..

[29] Aremntias S.L.D., Real J.C.D., Paz E., Dunne N. (2020). Advances in Biodegradable 3D Printed Scaffolds with Carbon-Based Nanomaterials for Bone Regeneration. Materials.

[30] Nambiar J., Jana S., Nandi S.K. (2022). Strategies for Emhancing Vascul arization of Biomaterial Based Scaffold in Bone Regeneration. Chem. Rec..

[31] Yang F., Li Y., Wang L., Che H., Zhang X., Jahr H., Wang L., Jiang D., Huang H., Wang J. (2024). Full-thickness osteochondral defect repair using a biodegradable bilayered scaffold of porous zinc and chondroitin sulfate hydrogel. Bioact. Mater..

[32] Yao R.H., Wang H., Shan R.F., Liu L., Zhao Y.Y., Sun Y.H., Yao X.H., Huang D., Hang R.Q. (2023). Biodegradable porous Zn-1Mg-3βTCP scaffold for bone defect repair: In vitro and in vivo evaluation. J. Mater. Sci. Technol..

[33] Zhou Y., Wu P., Yang Y., Gao D., Feng P., Gao C., Wu H., Liu Y., Bian H., Shuai C. (2016). The microstructure, mechanical properties and degradation behavior of laser-melted MgSn alloys. J. Alloys Compd..

[34] Xiwei L., Jianke S., Yinghong Y., Feiyu Z., Zhongjie P., Li L., Yufeng Z. (2015). Microstructure, mechanical properties, in vitro degradation behavior and hemocompatibility of novel Zn-Mg-Sr alloys as biodegradable metals. Mater. Lett..

[35] Su Y., Fu J., Du S., Georgas E., Qin Y.X., Zheng Y., Zhu D. (2022). Biodegradable Zn-Sr alloys with enhanced mechanical and biocompatibility for biomedical applications. Smart Mater. Med..

[36] Vojtech D., Kubasek J., Serak J., Novak P. (2011). Mechanical and corrosion properties of newly developed biodegradable Zn-based alloys for bone fixation. Acta Biomater..

[37] Wu S., Liu X., Yeung K.W., Liu C., Yang X. (2014). Biomimetic porous scaffolds for bone tissue engineering. Mater. Sci. Eng. R Rep..

[38] Kim J.S. (2018). Deep vein thrombosis prophylaxis after total hip arthroplasty in Asian patients. Hip Pelvis.

[39] Hybasek V., Kubasek J., Capek J., Alferi D., Pinc J., Jiru J., Fojt J. (2021). Influence of model environment complexity on corrosion mechanism of biodegradable zinc alloys. Corros. Sci..

[40] Mostaed E., Sikora-Jasinska M., Drelich J.W., Vedani M. (2018). Zinc-based alloys for degradable vascular stent applications. Acta Biomater..

[41] Capek J., Jablonska E., Lipov J., Kubatik T.F., Vojtech D. (2018). Preparation and characterization of porous zinc prepared by spark plasma sintering as a material for biodegradable scaffolds. Mater. Chem. Phys..

[42] Chuah L.F., Chew K.W., Bokhari A., Mubashir M., Show P.L. (2022). Biodegradation of crude oil in seawater by using a consortium of symbiotic bacteria. Environ. Res..

[43] Hou Y., Jia G., Yue R., Chen C., Pei J., Zhang H., Yuan G. (2018). Synthesis of biodegradable Zn-based scaffolds using NaCl templates: Relationship between porosity, compressive properties and degradation behavior. Mater. Charact..

[44] Jain D., Pareek S., Agarwala A., Shrivastava R., Sassi W., Parida S.K., Behera D. (2021). Effect of Exposure Time on Corrosion Behavior of Zinc-Alloy in Simulated Body Fluid Solution: Electrochemical and Surface Investigation. J. Mater. Res. Technol..

[45] Castro Y., Durán A. (2019). Control of Degradation Rate of Mg Alloys Using Silica Sol–Gel Coatings for Biodegradable Implant Materials. J. Sol-Gel Sci. Technol..

[46] Yang Y., Yuan F., Gao C., Feng P., Xue L., He S., Shuai C. (2018). A combined strategy to enhance the properties of Zn by laser rapid solidification and laser alloying. J. Mech. Behav. Biomed. Mater..

[47] Liu L., Meng Y., Dong C., Yan Y., Volisky A.A., Wang L.N. (2018). Initial formation of corrosion products on pure zinc in simulated body fluid. J. Mater. Sci. Technol..

[48] Wtroba M., Bednarczyk W., Szewczyk P.K., Kawałko J., Mech K., Grünewald A., Unalan I., Taccardi N., Boelter G., Banzhaf M. (2023). In vitro cytocompatibility and antibacterial studies on biodegradable Zn alloys supplemented by a critical assessment of direct contact cytotoxicity assay. J. Biomed. Mater. Res. Part B Appl. Biomater..

[49] Mostaed E., Sikora-Jasinska M., Mostaed A., Loffredo S., Demir A.G., Previtali B., Vedani M. (2016). Novel Zn-based alloys for biodegradable stent applications: Design, development and in vitro degradation. J. Mech. Behav. Biomed. Mater..

[50] Capek J., Kubasek J., Pinc J., Fojt J., Krajewski S., Rupp F., Li P. (2021). Microstructural, mechanical, in vitro corrosion and biological characterization of an extruded Zn-0.8 Mg-0.2 Sr(wt%) as an absorbable material. Mater. Sci. Eng. C.

[51] Ke G.Z., Yue R., Huang H., Kang B., Zeng H., Yuan G.Y. (2020). Effects of Sr addition on microstructure, mechanical properties and in vitro degradation behavior of as-extruded Zn−Sr binary alloys. Trans. Nonferrous Met. Soc. China.

[52] Yuan W., Xia D., Wu S., Zheng Y., Guan Z., Rau J.V. (2022). A review on current research status of the surface modification of Zn-based biodegradable metals. Bioact. Mater..

[53] Dai Q., Peng S., Zhang Z., Liu Y., Fan M., Zhao F. (2021). Microstructure and mechanical properties of zinc matrix biodegradable composites reinforced by graphene. Front. Bioeng. Biotechnol..

[54] He J., Fang J., Wei P., Li Y., Guo H., Mei Q., Ren F. (2021). Cancellous bone-like porous Fe@Zn scaffolds with core-shell-structured skeletons for biodegradable bone implants. Acta Biomater..

[55] Kubásek J., Vojtěch D., Jablonska E., Pospíšilová I., Lipov J., Ruml T. (2016). Structure, mechanical characteristics and in vitro degradation, cytotoxicity, genotoxicity and mutagenicity of novel biodegradable Zn–Mg alloys. Mater. Sci. Eng. C.

[56] Sivaraj D., Arumugam G., Kalimuthu V., Rajendran R. (2022). Enhanced anti-biofilm and biocompatibility of Zn and Mg substituted β-tricalcium phosphate/functionalized multiwalled carbon nanotube composites towards A. baumannii and Methicillin-Resistant Staphylococcus aureus, and MG-63 cells. Int. J. Pharm..

[57] Hernández-Escobar D., Champagne S., Yilmazer H., Dikici B., Boehlert C.J., Hermawan H. (2019). Current status and perspectives of zinc-based absorbable alloys for biomedical applications. Acta Biomater..

[58] Guyton A.C. (2015). Textbook of Medical Physiology e-Book.

[59] Esmaily M., Svensson J.E., Fajardo S., Birbilis N., Frankel G.S., Virtanen S., Johansson L.G. (2017). Fundamentals and advances in magnesium alloy corrosion. Prog. Mater. Sci..

